# Classification of ischemia from myocardial polar maps in ^15^O–H_2_O cardiac perfusion imaging using a convolutional neural network

**DOI:** 10.1038/s41598-022-06604-x

**Published:** 2022-02-18

**Authors:** Jarmo Teuho, Jussi Schultz, Riku Klén, Juhani Knuuti, Antti Saraste, Naoaki Ono, Shigehiko Kanaya

**Affiliations:** 1 Data Science Center, Nara University of Science and Technology, Nara, Japan; 2 Turku PET Centre, University of Turku, Turku, Finland; 3 Turku PET Centre, Turku University Hospital, Turku, Finland; 4 Heart Centre, Turku University Hospital and University of Turku, Turku, Finland; 5 Department of Science and Technology, Nara University of Science and Technology, Nara, Japan

**Keywords:** Molecular imaging, Radionuclide imaging, Cardiology, Information technology, Medical imaging

## Abstract

We implemented a two-dimensional convolutional neural network (CNN) for classification of polar maps extracted from Carimas (Turku PET Centre, Finland) software used for myocardial perfusion analysis. 138 polar maps from ^15^O–H_2_O stress perfusion study in JPEG format from patients classified as ischemic or non-ischemic based on finding obstructive coronary artery disease (CAD) on invasive coronary artery angiography were used. The CNN was evaluated against the clinical interpretation. The classification accuracy was evaluated with: accuracy (ACC), area under the receiver operating characteristic curve (AUC), F1 score (F1S), sensitivity (SEN), specificity (SPE) and precision (PRE). The CNN had a median ACC of 0.8261, AUC of 0.8058, F1S of 0.7647, SEN of 0.6500, SPE of 0.9615 and PRE of 0.9286. In comparison, clinical interpretation had ACC of 0.8696, AUC of 0.8558, F1S of 0.8333, SEN of 0.7500, SPE of 0.9615 and PRE of 0.9375. The CNN classified only 2 cases differently than the clinical interpretation. The clinical interpretation and CNN had similar accuracy in classifying false positives and true negatives. Classification of ischemia is feasible in ^15^O–H_2_O stress perfusion imaging using JPEG polar maps alone with a custom CNN and may be useful for the detection of obstructive CAD.

## Introduction

Recently, it has become more evident that cardiovascular imaging, and, in particular, nuclear cardiology including single photon emission computed tomography (SPECT) and positron emission tomography (PET) myocardial perfusion imaging can benefit from implementation of machine learning methods and artificial intelligence^1,2^. Deep Learning is of special interest for nuclear cardiology as it offers the possibility to directly process cardiac images to identify and characterize myocardial ischemia^1^.

Historically, the earliest study investigating the use of machine learning algorithms for myocardial perfusion imaging (MPI) was performed by Fujita et al.^3^, who used a three-layer feed-forward neural network to classify polar plots from 74 polar maps with Thallium-201 SPECT. The network showed promising results, although improvements were needed to improve the classification performance up to level of experienced radiologist. Thereafter, the first approaches using support vector machine (SVM)^4^ and LogitBoost^5^ algorithms combined with information extracted from polar maps were used in integrating quantitative perfusion and functional variables for prediction of coronary artery disease. The first modern study to use SPECT polar maps alone with artificial neural networks was performed by Nakajima et al.^6^.

More recently, a deep learning model was used to classify subjects from a large SPECT study performed with ^99m^Tc-sestamibi or ^99m^Tc-tetrofosmin MPI. Polar maps of 1638 subjects were classified with reference data from invasive coronary angiography (ICA)^7^. The method was later extended to semiupright and supine stress MPI from 1160 patients undergone ^99m^Tc-sestamibi MPI^8^. The study showed that deep learning improved automatic prediction of obstructive coronary artery disease (CAD) compared with the current standard clinical method. In another SPECT study, Spier et al.^9^ compared several network architectures to classify 946 labeled polar maps with relatively good agreement with a human observer.

For PET imaging, Togo et al. applied a deep learning model as a feature extractor in combination of a SVM classifier to classify polar maps from 85 subjects undergone 18F-FDG PET^10^. In comparison against SUV-based classification, the machine learning approach showed improved performance. Recently, Juarez-Orozco et al.^11^ applied deep learning to classify quantitative polar maps from N-13 perfusion study using data from 1185 patients, where the deep learning model outperformed 4 comparator models used for classification of adverse cardiac events. The authors thus concluded that using DL is feasible in the direct evaluation of quantitative PET myocardial perfusion polar maps.

As seen above, most of the approaches have been conducted using SPECT data, with only a few studies performed on PET data, and especially using the information available from quantitative PET perfusion polar maps. In a recent review, the authors even stated that cardiovascular imaging, especially nuclear cardiology and PET myocardial perfusion imaging, comprises a particular niche for the implementation of artificial intelligence (AI)^1^. Moreover, there are multitude of potential applications where AI could be beneficial in the area of nuclear cardiology^12^. Thus, it is especially important to investigate the application of machine learning methods in PET myocardial perfusion imaging.

For perfusion PET imaging, these methods are especially of interest. PET imaging using ^15^O–H_2_O MPI allows to assess myocardial perfusion with absolute quantitation of myocardial blood flow (MBF) at rest and during stress, where local reduction of regional stress MBF or myocardial flow reserve indicates the presence of ischemic disease with tracer-specific cut-off thresholds^13,14^. ^15^O–H_2_O MPI is performed using measurement of tracer concentration during dynamic PET acquisition, followed by image analysis and kinetic modeling via software^15^. The results are visualized in a form of polar plot, which shows the territorial division of MBF in the apex and three coronary artery territories, namely the: right coronary artery (RCA), left anterior descending (LAD) and left circumflex (LCx) coronary artery. ^15^O–H_2_O MPI is increasingly used in evaluation of patients with suspected CAD and accurately detects obstructive CAD based on invasive angiography and fractional flow reserve (FFR) measurements^13,16^. Thus, a natural continuation of the previous works would be to apply machine learning in classification of polar maps of stress MBF derived from ^15^O–H_2_O MPI in order to detect obstructive CAD.

The aim of this study was to implement an automatic classifier based on a custom two-dimensional (2D) convolutional neural network (CNN) architecture to classify polar maps of stress MBF created with Carimas 2.9 software as ischemic or non-ischemic in ^15^O–H_2_O myocardial perfusion imaging. To our knowledge, this is the first time a custom CNN is used to classify polar maps in ^15^O–H_2_O perfusion imaging.

## Materials and methods

### Study population

The patient population consisted of subjects with suspected obstructive CAD. The population included prospectively recruited subjects during 2007–2011 in Turku University Hospital with stable chest pain or equivalent symptom and intermediate pre-test probability of obstructive CAD. The study of Stenström et al.^17^ is referred for additional details. From the original 189 subjects in the study, subjects with readily available ICA and PET perfusion data from the stress imaging study were included, resulting in total of 138 subjects. From the subjects, 73 were males and 65 were females, with age (mean ± SD) of 66 ± 7 years. There were in total of 56 patients with obstructive CAD in at least one epicardial coronary artery defined by ICA of whom 25 were in the training, 11 in the validation and 20 in the test cohorts. The study was approved by the local ethics committee of the Hospital District of Southwest Finland and was conducted according to the guidelines of the Declaration of Helsinki. Informed consent was collected from all patients.

### PET/CT image acquisition

Patients were scanned with the Discovery VCT PET/CT scanner (GE Healtchare, US) as previously described in^13,17,18^. The study protocol included computed tomography coronary angiography (CTA) and myocardial PET perfusion imaging with PET/CT hybrid scanner. An adenosine stress perfusion PET was performed immediately after CT-based attenuation correction. Adenosine was started 2 min before the start of the scan and was infused at 140 μg/kg body weight per minute. 15O-labeled water (900 to 1100 MBq) was injected (Radiowater Generator, Hidex Oy, Finland) as an intravenous bolus over 15 s. A dynamic acquisition of the heart was performed (14 × 5 s, 3 × 10 s, 3 × 20 s, and 4 × 30 s). Images were reconstructed using two-dimensional ordered expectation maximization algorithm (2D-OSEM) using a 35 cm field of view, 128 × 128 matrix size, 2 iterations, 20 subsets and a 6.0 mm Gaussian post-filter.

### Invasive coronary artery angiography

Coronary angiograms were performed on Siemens Axiom Artis coronary angiography system (Siemens, Erlangen, Germany) as described earlier. Measurement of FFR was performed for stenoses with intermediate severity (30–80%) when feasible to assess their hemodynamic significance. Quantitative analysis of coronary angiograms was performed using software with automated edge detection system (Quantcore, Siemens, Munich, Germany) by an experienced reader. Obstructive CAD was defined as either $\ge$ 50% stenosis on ICA or FFR < 0.8. When FFR was available, stenoses with FFR $\ge$ 0.8 were classified as non-significant, regardless of the degree of coronary narrowing.

### Analysis of the cardiac perfusion data

Dynamic PET perfusion images were quantitatively analyzed with Carimas 2.9 software (Turku PET Centre, Finland) by a single reader (JS) blinded to the ICA results. For the analyses, the orientation of the heart was identified manually, after which the myocardium was automatically detected by the software. The resulting regions-of-interest (ROIs) were manually adjusted if necessary. Mathematical modelling based on a single tissue compartment model was performed to yield quantitative polar maps of stress MBF values in units of ml/g/min^15^. Based on previously defined threshold for ischemic stress MBF (< 2.3 ml/g/min)^13^, stress MBF values in polar maps were uniformly scaled from 0 to 3.5 ml/g/min using Rainbow color scale.

### Polar map and label dataset description and pre-processing

A total of 138 polar maps of stress MBF with corresponding ICA labels were used in the evaluation. Data from ICA was used as a gold standard for assigning the reference labels. Based on obstructive CAD defined by ICA, each polar map was classified as ischemic (1) or non-ischemic (0). Thus, the classification of polar maps then becomes a binary classification problem.

**Figure 1:**
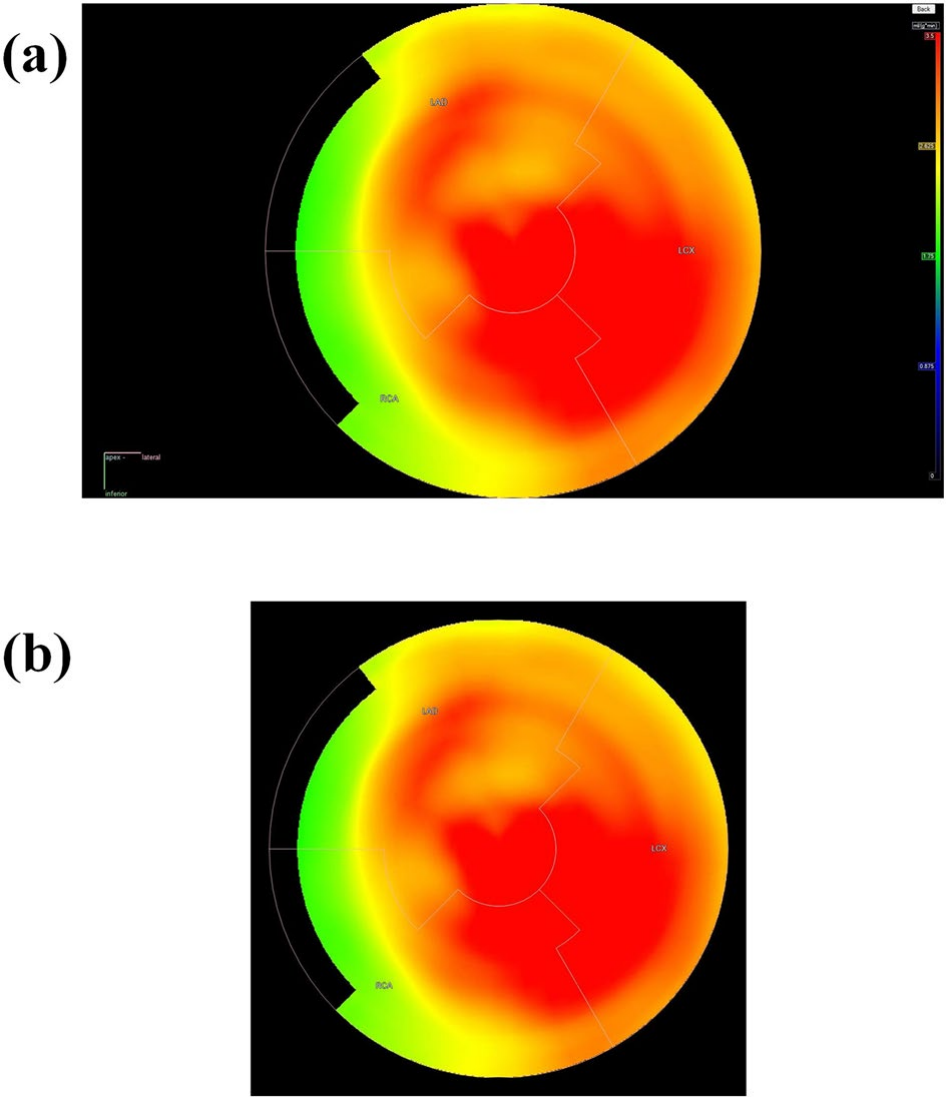
An example of an (**a**) original polar map JPEG image before cropping and resizing to smaller dimensions. After cropping and resizing, (**b**) only the polar map and the superimposed regional markings signifying LAD, LCX and RCA remain in a rectangular shape of 256 × 256 pixels. All polar maps were scaled between 0 and 3.5 ml/g/min before image export, where red and yellow color indicate normal stress MBF, whereas green and blue color indicate reduced stress MBF.

Polar maps were exported as high-resolution two-dimensional JPEG images from Carimas, with original image size of 1024 × 1024 pixels. The images were cropped automatically using a predefined rectangular region to remove the majority of black background, followed by visual inspection of the quality of the cropping. An example of the original polar map and a cropped polar map can be found from Fig. 1. In the processing pipeline, the cropped polar map is reduced into a size of 256 × 256 pixels and the pixel values were scaled between 0 and 1. Thus, the polar maps were normalized between [0,1]. All three color channels (R,G,B) were used as an input. Each polar map contained a corresponding label as .txt file with was determined based on the reference ICA data (1 = ischemic, 0 = non-ischemic).

### 2D CNN architecture

The following software were used, with the version numbers in parenthesis, python (2.7.17), keras (2.3.1), tensorflow (1.14.0), scipy (1.2.2), numpy (1.16.6), matplotlib (2.2.4), pandas (0.24.2) and sklearn (0.20.4)^19–26^.

A custom 2D CNN was built with 4 layers of convolutional filters using keras and tensorflow. The final network architecture was fixed after a systematic search of different combination of hyperparameters, convolutional layers and inclusion or exclusion of pooling operations. In addition to the previous, we also evaluated on using kernel regularization on all the network layers versus final layer only. The best model was selected on the basis of stability and performance on the on the training and validation data and the final performance evaluation on the test dataset. The network architecture selection is discussed in detail in the Discussion section, under paragraph “Network design aspects”.

**Figure 2:**
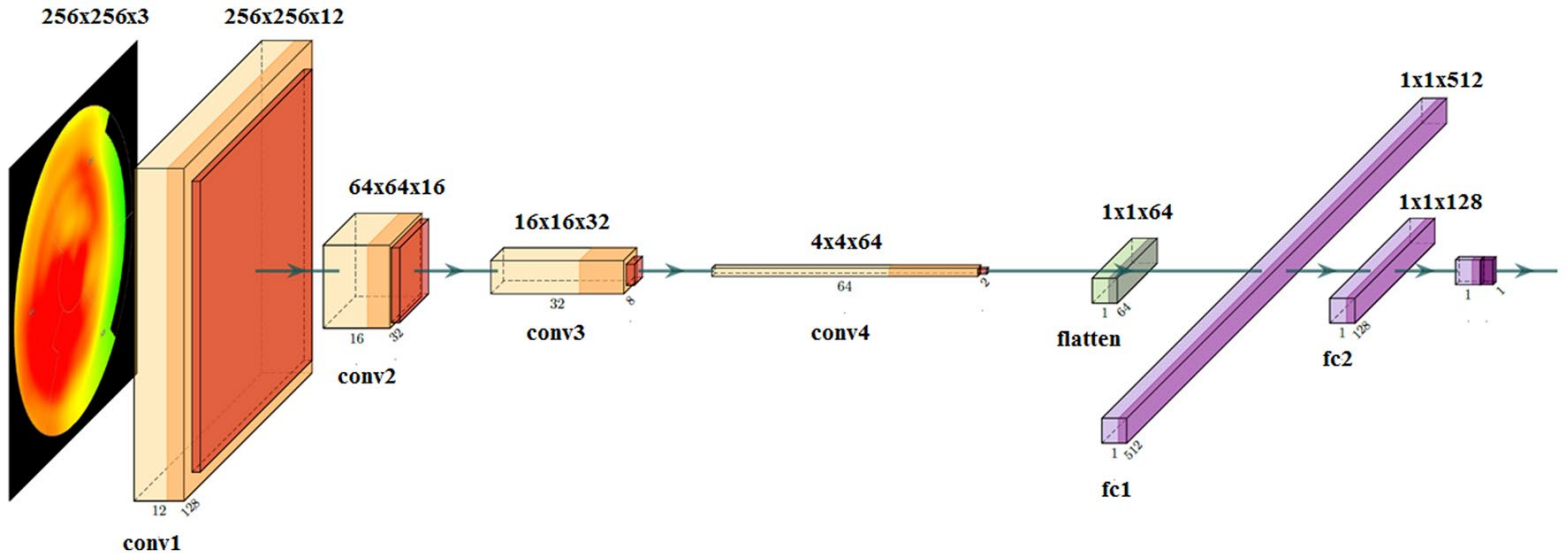
The 2D CNN architecture used in this study. The input polar map is a 256 × 256 RGB image. Each 2D convolutional layer contains a 3 × 3 kernel with stride 2 × 2 and ReLU as activation function. Filter sizes are increased gradually from 12, 16, 32 to 64. Each convolutional layer is followed by a max pooling layer with a window size of 2 × 2. Thus, the output of the last convolution and max-pooling layer is reduced to 1 × 1 × 64, which is given as an input to the flatten layer. The last layers contain a flatten layer of size 64 followed by two dense layer of sizes 512 and 128, using ReLU as activation function. The output layer is a dense layer with 1 output (0 or 1) with a sigmoid function. The input size of each convolutional layer and the flatten layer is given above each layer. For the fully connected layers, the output size is given. conv = 2D convolutional layer, flatten = flatten layer, fc = fully connected layer.

The final network architecture is shown in Fig. 2. A sequential model was used with four 2D convolutional layers each followed by a 2D max pooling operation with 2 × 2 window. The number of filters for each convolutional layer was increased gradually as: 12, 16, 32 and 64. The kernel size was set as 3 × 3, whereas strides were set as 2 × 2 for all layers. A rectilinear (ReLU) activation function was used or each layer. Kernel regularization was used for the last output layer using L2-norm, with penalty set to 0.1.

After the last max pooling layer, a flatten operation was used and the output was fed to two consecutive fully-connected layers where the first layer had 512 nodes and the last layer had 128 nodes. Both fully connected layers used a ReLU for the activation function. The last layer is a dense layer consisting of one binary output (0 for non-ischemic, 1 for ischemic) and uses a sigmoid activation function.

A stochastic gradient descent was used for the optimizer, with learning rate of 0.005, decay of 1e-8 and momentum of 0.9. Binary cross entropy was used as the loss function whereas the area under the receiver operating characteristic curve (AUC) was selected as the metric to be optimized. For comparison, we also evaluated using binary accuracy (ACC) as the optimization metric, as this is commonly used for binary classification.

During the network training, the training data was shuffled before each epoch. Batch size of 20 and 35 epochs were used. After 35 epochs, the classification performance was not improved. Class weights were used to weight the model by setting weights as 1 for non-ischemic and 3 for ischemic. The final selection of the hyperparameters were decided on the performance on the training and validation data as described in the section below.

### Training, validation and testing procedures

**Figure 3:**
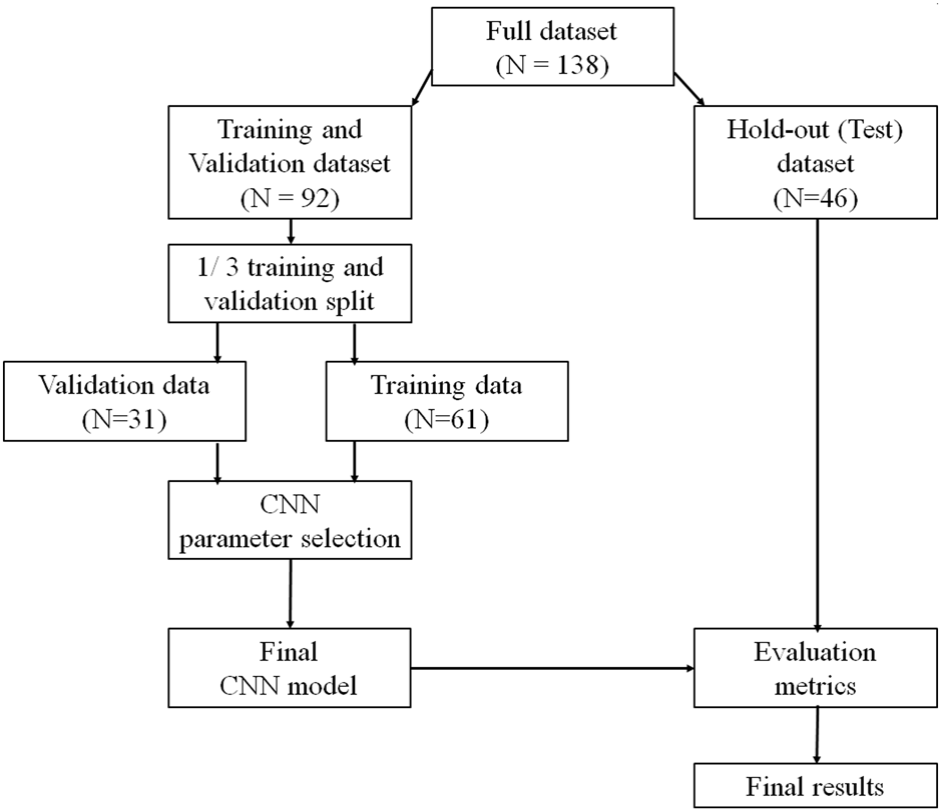
Workflow diagram of the analysis pipeline used to select the optimum parameters for the CNN and to evaluate the performance of the final model. The training, validation and test steps were repeated 100 times for the CNN to evaluate the model stability.

A workflow image from the whole analysis pipeline can be found from Fig. 3. The validation split option was set to 1/3, resulting to total of 61 patients used for training and 31 used for validation for the appropriate selection of the parameters of the CNN. A separate hold-out dataset was used as a test set to evaluate the final performance of the CNN model which was fixed after training and validation. This test dataset contained 46 patients. Patients were split randomly into the datasets.

A chi-squared test was used for independence testing between the training, validation and test dataset labels. The test resulted in *p* > 0.05 for between the dataset labels, thus it was concluded that the datasets were independent of each other. All statistical testing was performed with R version 4.0.2^27^.

During the training, the resized polar map was given as input with the corresponding reference label. For the validation and testing, the trained network was given only the resized polar map for prediction and the accuracy of the predicted label was compared against the reference label. The predicted label is binary for the CNN, either ischemic (1) or non-ischemic (0). This predicted label was then compared to the reference ICA label from each patient.

For the CNN, all the steps including training/validation and testing were run 100 iterations by only varying the seed in the random number generator. At each iteration, the predicted classes with the reference labels for each run was saved to evaluate the network stability. Thereafter, the classification performance was evaluated for both CNN and for the clinical interpretation of the polar maps using commonly applied classification evaluation metrics.

### Comparison to the clinical interpretation of the polar maps

For the 46 subjects included in the test dataset, the clinical end-point classification assessed via clinical interpretation of the polar maps was used as a reference metric to compare the performance of the CNN. The clinical classification includes the visual inspection of the polar maps and the use of a previously defined threshold for ischemic stress MBF (< 2.3 ml/g/min)^13^. The classification results from the clinical interpretation were rated as ischemic (1) if a clinically significant reduction in the PET perfusion was detected and (0) if no clinically significant reduction in perfusion was found. Thereafter, the classification accuracy of the clinical interpretation and CNN were compared using the metrics specified below.

### Classification accuracy metrics

The following metrics were used in the evaluation of the CNN performance for both the training/validation and test dataset: accuracy (ACC), area under the receiver operating characteristic curve (AUC), F1 score (F1S), sensitivity (SEN), specificity (SPE) and precision (PRE). AUC was calculated as the area under the receiver-operating characteristic curve.

In addition to these metrics, we also calculated the net benefit^28^ to evaluate the CNN performance. The net benefit was visualized in a decision curve containing the net benefit value over threshold probability values from 0 to 100%.

All data visualization and analysis were performed with MATLAB v2017a (Mathworks Inc., US) based on the predicted binary labels and reference CAD labels. We report all classification accuracy metrics for the CNN using AUC as the optimization metric in the Results section below and using ACC as the optimization metric in the Supplementary Data. We report the values for ACC, F1S, SEN, SPE and PRE in addition to true positives (TP), true negatives (TN), false positives (FP) and false negatives (FN) as median with the interquartile range over the 100 runs for the data with the CNN.

All of the metrics specified above were also calculated for the clinical classification results to create a reference of performance for the CNN. In addition, we investigated the agreement between the CNN and the clinical interpretation in classifying the subjects into the categories of ischemic, TP, TN, FP and FN, in cases where the CNN labeled a subject to a certain category over 50 times out of 100 runs. The agreement was investigated by calculation of Cohen’s Kappa coefficient $\kappa$. We report the value of $\kappa$ between the CNN and the clinical interpretation for ischemic cases, TP, TN, FP and FN, respectfully.

The equations for calculation of all the classification accuracy metrics are given in Supplementary Data 1.

## Results

**Figure 4:**
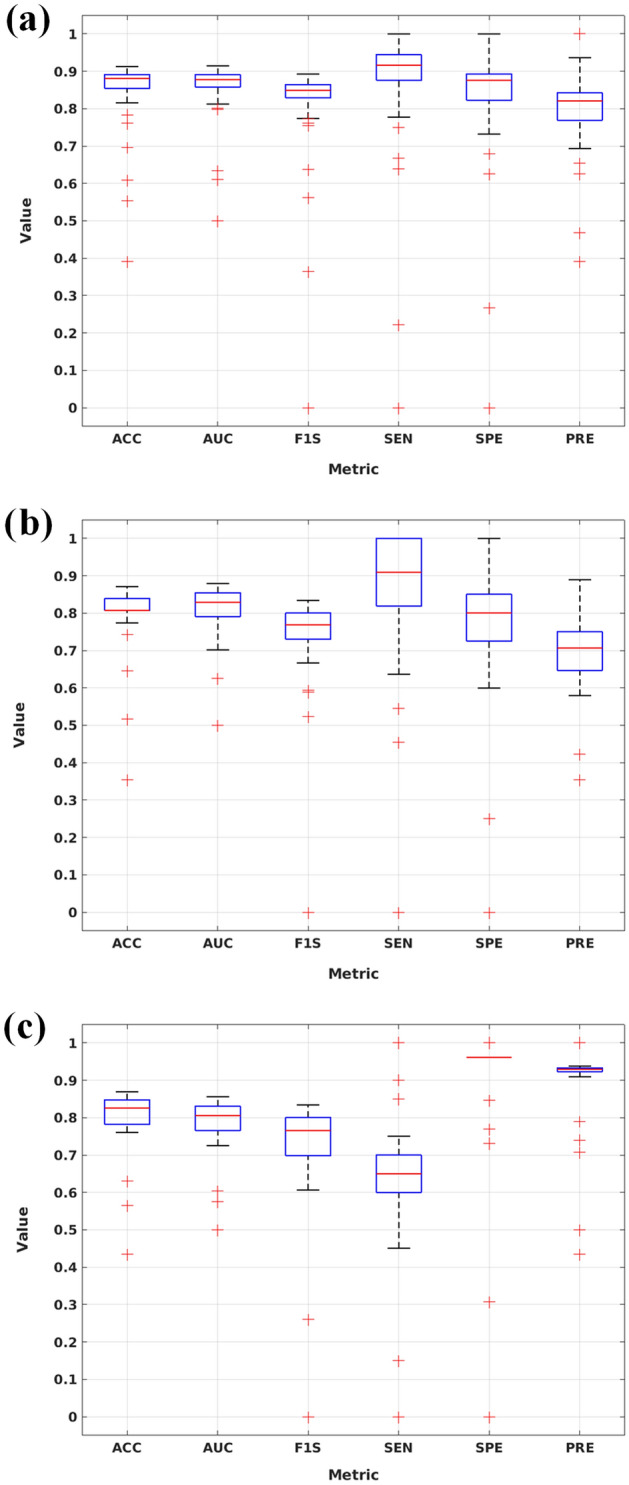
CNN classification accuracy with (**a**) training (**b**) validation and (**c**) test data. ACC = accuracy, AUC = area under the receiver operating characteristic curve, F1S = F1 score, SEN = sensitivity, SPE = specificity, PRE = precision.

Figure 4 contains the evaluation of classification performance using the training, validation and test data with different metrics over 100 runs of the data.

Table 1 contains the amount of TP, TN, FP and FN with median and interquartile range of the CNN on 100 runs of the test data in addition to the results with the clinical interpretation. The classification results of the CNN and the clinical interpretation differed only in two cases, which the CNN labeled as false negative instead of true positive. The CNN and the clinical interpretation agreed fully on the FP and TN cases.

**Table 1:** The amount of true positives (TP), true negatives (TN), false positives (FP) and false negatives (FN) with median and interquartile range (IQR) of the CNN on 100 runs of the data in addition to the results with the clinical interpretation with the hold-out (test) dataset.

Method	TP	TP IQR	TN	TN IQR	FP	FP IQR	FN	FN IQR
CNN	13	2	25	0	1	0	7	2
Clinical	15	N/A	25	N/A	1	N/A	5	N/A

Table 2 contains the classification accuracy evaluation metrics with median and interquartile range of the CNN classification performance over 100 runs of the test data. In addition, the results with the clinical interpretation using the test data are presented for comparison. The CNN and the clinical interpretation had small differences in performance for the majority of the metrics.

**Table 2:** Classification accuracy evaluation metrics with median and interquartile range (IQR) in parenthesis of the CNN classification performance over 100 runs of the data in addition to the results with the clinical interpretation with the hold-out (test) dataset.

Method	ACC	AUC	F1S	SEN	SPE	PRE
CNN	0.8261 (0.0652)	0.8058 (0.0654)	0.7647 (0.1014)	0.6500 (0.1000)	0.9615 (0.0000)	0.9286 (0.0103)
Clinical	0.8696	0.8558	0.8333	0.7500	0.9615	0.9375

Table 3 contains the agreement of the CNN and the clinical interpretation in classifying subjects to different categories. The CNN and the clinical interpretation had good agreement on the ischemic subjects, TP and FN cases and very good agreement on the amount of TN and FP cases. The lowest agreement was in FN cases.

**Table 3:** Cohen’s Kappa ($\kappa$) calculated for the CNN and the clinical interpretation indicating agreement between classification of the subjects to categories of ischemic, TP, TN, FP and FN.

	Ischemic	TP	TN	FP	FN
$\kappa$	0.8026	0.7951	1.000	1.000	0.6183

Good agreement is reached in all categories. The lowest agreement was reached in subjects labeled as FN.

**Figure 5:**
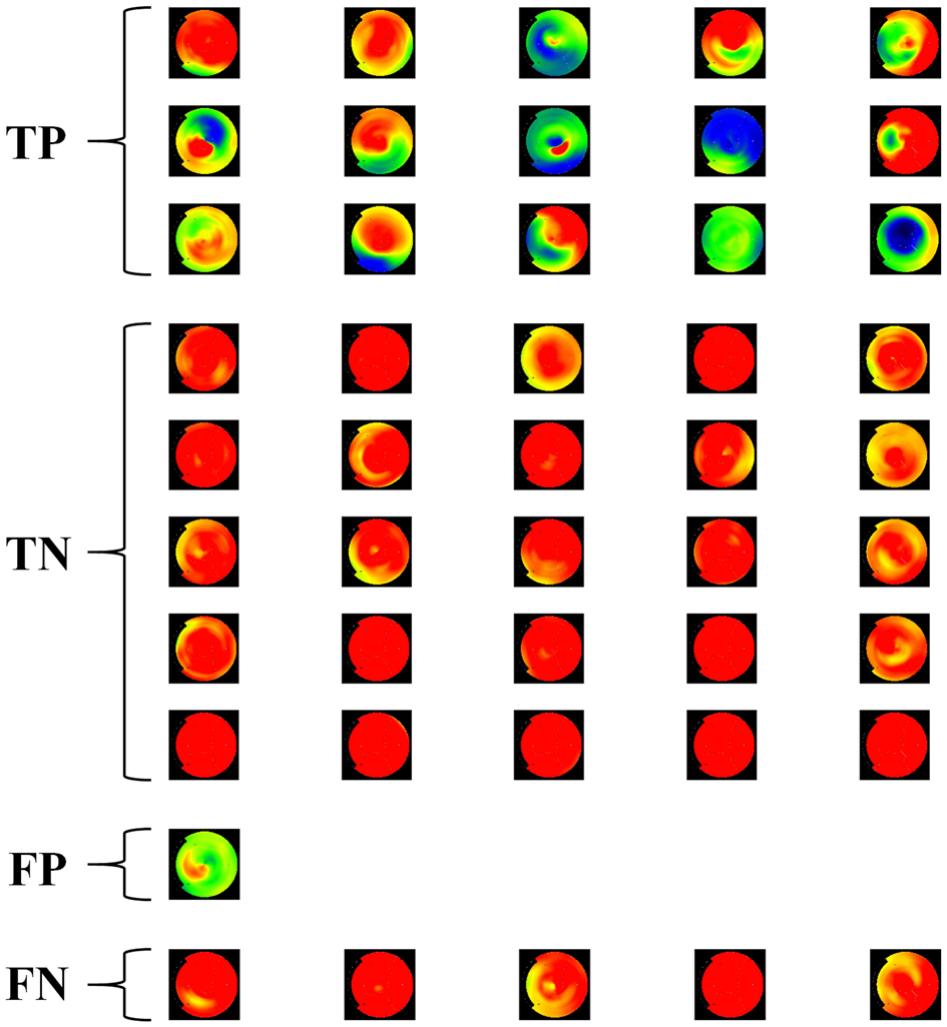
Visualization of polar maps classified as true positive (TP), true negative (TN), false positive (FP) and false negative (FN) from the best classification result with the CNN (accuracy 0.8696, matching the clinical interpretation).

Figure 5 contains a visualization of the true positive, true negative, false positive and false negative polar maps of the best classification result in the test data by the CNN (accuracy 0.8696). A total of 15 TP, 25 TN, 1 FP and 5 FN cases were detected by the network, which corresponded to the classification results of the clinical interpretation.

**Figure 6:**
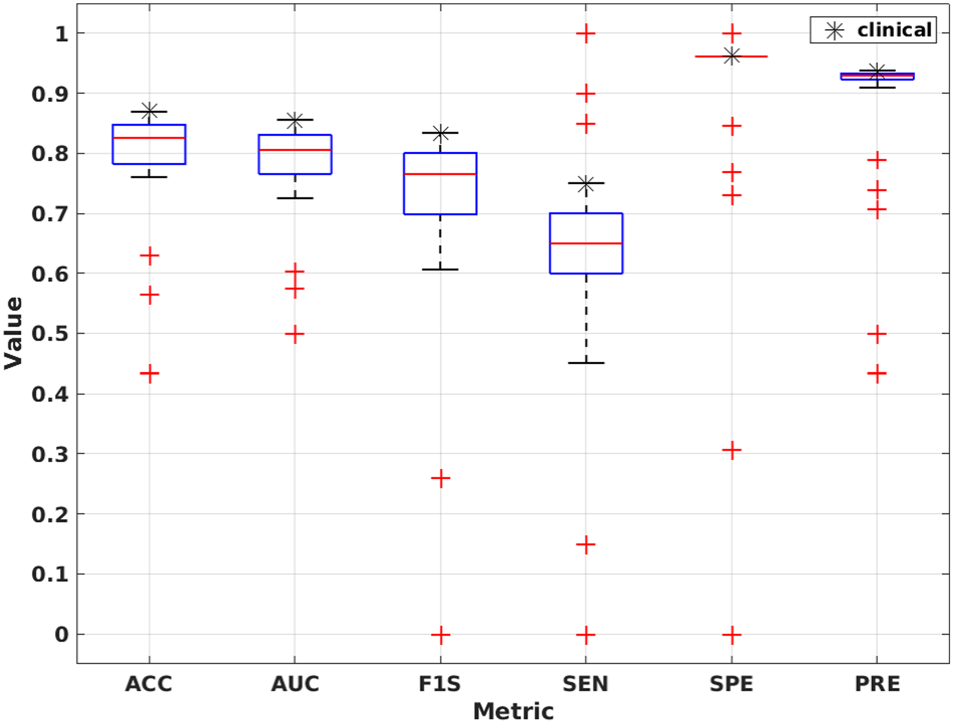
CNN classification performance from 100 runs of the network versus the clinical interpretation. The best classification results of the CNN match the performance of clinical interpretation ACC = accuracy, AUC = area under the receiver operating characteristic curve, F1S = F1 score, SEN = sensitivity, SPE = specificity, PRE = precision.

Figure 6 contains a comparison plot of the CNN classification performance against the clinical interpretation using the test dataset. As can be seen, the maximum observed classification result of the CNN matches the one from the clinical interpretation in majority of the metrics although the CNN median performance is lower.

**Figure 7:**
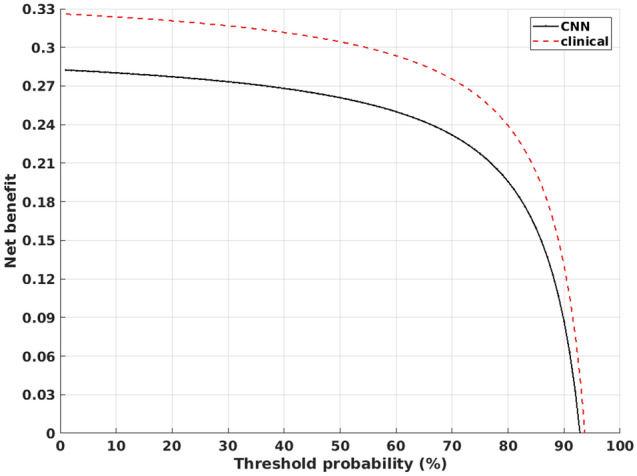
Decision curve with net benefit for all threshold probabilities from 0 to 100% for CNN median over 100 runs and the clinical interpretation using the test data. Only positive values in the range of 0 to 1 for the net benefit are shown.

Figure 7 contains the decision curve containing the net benefit calculated as the median net benefit over all 100 runs of data with the CNN and net benefit for the clinical interpretation from the test data. The net benefit is shown all threshold probabilities from 0 to 100%.

## Discussion

We’ve shown that using a custom 2D CNN is feasible for detection of ischemia based on exported polar map JPEG images of stress MBF alone in ^15^O–H_2_O MPI. Overall, the network achieved decent performance for the evaluated parameters, despite using a small dataset (<150 subjects). This was also the first time when a custom 2D CNN was applied to classify ischemia in ^15^O–H_2_O perfusion imaging. Altogether, these results show promise for further evaluation and development of the proposed approach, as the CNN and the clinical interpretation resulted in classifying only 2 cases differently.

### Evaluation of network performance

Overall, the CNN showed good performance over all evaluation metrics with relatively good stability over 100 runs (Fig. 4). The amount of cases classified as TN or FP was similar between the CNN and the clinical interpretation (Table 1). The CNN differed from the clinical interpretation in 2 cases, which were labeled as false negatives by the CNN. These cases were classified into false negative category systematically, as the CNN labeled them 90 times and 88 times out of 100 runs as false negatives. The polar maps for these cases are given in Supplementary Data 2, Supplementary Fig. S1, showing that both cases are challenging to classify visually without access to the quantitative values.

In addition, we achieved a median accuracy of 0.83, AUC of 0.81, F1 score of 0.76 with sensitivity and specificity of 0.65 and 0.96, respectively (Table 2). From these metrics, the ACC, AUC, SPE and PRE can be considered to be very comparable between the CNN and the clinical interpretation. However, the performance of the CNN could be further improved in F1S and SEN. The largest difference compared to the clinical interpretation can be seen in the CNN sensitivity with a value of 0.65 versus a value of 0.75 achieved by the clinical interpretation, warranting for improvement.

There was a good agreement between the CNN and the clinical interpretation in classifying cases to ischemic, TN and FP categories by the CNN (Table 3). However, the CNN and the clinical interpretation showed the lowest agreement on the FN cases, with $\kappa$ value of 0.62. This would indicate that the major difference in CNN performance to the clinical interpretation is most attributed by the classification of FN cases, which can be seen in the classification of the two challenging cases (Supplementary Fig. S1). Still, if more of these challenging borderline cases would be available for training, the FN rate could probably be further reduced.

Moreover, the CNN had a very low variance in classification of false positives in the test set, as only one case was classified consistently as false positive, giving very low variation in SPE (Fig. 4). This reflects the performance of the clinical interpretation. Both the CNN and the clinical interpretation classified only one subject as false positive, which was the same subject between the CNN and the clinical interpretation.

The CNN achieving the best classification accuracy reached also the exact classification results as the clinical interpretation (Fig. 5). In this case, the amount of FN between the CNN and the clinical interpretation was identical, reaching 5 cases. Some of these FN cases in (Fig. 5) could be explained by the difference in methodology between CAD and PET perfusion imaging, that is, an anatomically significant stenosis detected in CAD does not always result in reduction of myocardial perfusion in PET. This would mean that the MBF value would be still remain in the normal range despite of a vessel blockage, making the stenosis hard to detect quantitatively or visually.

The median performance was lower for the CNN than that of the clinical interpretation (Fig. 6). However, the best classification result from the CNN matches well that of the clinical interpretation. Although these results show promise for the application for the CNN, there is still room for improvement in the classification accuracy.

The CNN achieved a net benefit value close to the clinical interpretation, however the clinical interpretation showed to be superior over all of the threshold probability values (Fig. 7). This would indicate that the CNN alone would not reach a comparable diagnostic performance versus the clinical interpretation over all the threshold probabilities. However, the best classification result with the CNN in (Fig. 5) reached the same net benefit as the clinical interpretation. None of the classification results over 100 runs was superior over the clinical interpretation.

In comparison to other studies, an accuracy of 0.82 ± 0.05 with sensitivity of 87%, specificity of 77% was achieved when classifying quantitative polar maps in N-13 perfusion PET was reported by Juarez-Orozco et al.^11^. In a large SPECT study, per-vessel sensitivities in the range of 0.59 to 0.77 and AUC of 0.74 to 77 were reported in^7^. In another study, Betancur et al.^8^ showed per-patient AUC of 0.81 and sensitivity of 65.6% to 84.8%. In comparison to the CNN model in^9^, which is closer to our approach concerning the model architecture, the authors reported an agreement of 0.83, sensitivity of 0.66 and specificity of 0.98. In this regard, we could achieve equal performance of the previously reported approaches.

Finally, we evaluated two classification metrics and noted that both using AUC and ACC did not result in difference of performance (Supplementary Data 3). The classification performance using the training, validation and test data with different metrics over 100 runs of the data are in Supplementary Fig. S2. The comparison versus the clinical interpretation and the net benefit with ACC are given in Supplementary Fig. S3 and S4, respectively. In short, with the exception of a few outlier values over the 100 runs, the performance between ACC and AUC could be considered identical.

### Network design aspects

We presented a custom 2D CNN, which was built from ground up for this classification task. The final model was selected after a systematic comparison of different architectures and found that this architecture performed the most stable with the training, validation and test datasets. In detail, we followed the approach descried in the article of Xue et al^32^, where a systematic hyperparameter search was conducted using different combination of parameters and layers and best model was selected on the basis of performance across the datasets.

During the network evaluation, we experimented with: (a) different amount of convolutional and deep layers, (b) various combination of different network settings, (c) inclusion or exclusion of max pooling operation and (d) implementing kernel regularization on different amount of layers. The results from these different CNN architectures are summarized in Supplementary Data 4, Supplementary Fig. S5. The net benefit was also worse for all methods presented in Supplementary Fig. S5, when compared to the selected CNN architecture in this paper.

The most optimal performance was achieved with 2 deep layers and 4 convolutional layers. Either increase or reduction of both kind of layers did not improve the performance. We also found that fixing the number of strides and kernel size over the convolutional layers improved the performance, compared to e.g. first layer having larger kernel size. The optimum performance was also achieved when using kernel regularization on the final layer only.

Moreover, we evaluated the network with and without max pooling operation and noticed that inclusion of max pooling clearly increased the network stability and performance over 100 runs of the data. Removing max pooling resulted in larger variance in accuracy over consecutive runs.

Of note is that the approaches used in the papers of Betancur et al.^7^ and Spier et al.^9^ both applied max pooling. Our network also uses considerably larger images (256 × 256) than the approaches in both papers (64 × 64 and 23 × 20), although the dimensionality of the polar map is reduced by both 2D convolution by 2 × 2 strides and max-pooling operations at each layer. Similarly to our study, in^11^, RGB color coded polar maps with a resolution of 223 × 223 pixels were used for analysis.

Finally, it should be noted that there are several freely available machine learning and CNN models which could be applied for image classification purposes, either with modifications or using them as backbones. The comparison of such approaches, an extensive study, should be performed systematically in the future.

### Future work and limitations

Our approach differs somewhat from the approaches used e.g. in^11^, as we apply only the JPEG image of the polar map for an input for the network, without any supporting quantitative or clinical information. This makes classification more challenging for the CNN as the network has no other supporting information than the image alone, as classification is performed solely on the visual appearance of the polar maps. On the other hand, in the clinical interpretation, the clinical observer does have at least the quantitative information at his/her disposal. Considering this difference, the CNN performed quite admirably as only two cases were classified differently by the CNN and as classified according to the clinical interpretation.

Our approach could be improved by importing the polar maps as raw DICOM export, as this export option was not available in the Carimas software at the time of this study. A raw DICOM export would preserve quantitative values and omit lossy compression used in JPEG. However, there is one advantage in our current methodology over raw export data. In clinical routine, the polar maps are saved as an attachment of an exam report and sent to PACS. Thus, our methodology could allow to perform classifications retrospectively based on the saved exam report images without an extensive reanalysis of the study.

In clinical reading, hybrid imaging data from coronary computed tomography angiography (CTA) is used to support the diagnostic decision in addition to patient-specific risk factors, which some are indicated in^17^. Our test data contained polar maps where the defects are hard to determine visually, making the classification problem more challenging. This can be seen when comparing the TN and FN cases (Fig. 4) and the maps systematically classified as FN contained in Supplementary Fig. S1. Potentially, integration of hybrid information from CT or MRI images could be used to improve the CNN accuracy, as suggested by Juarez-Orozco et al.^29^.

Furthermore, other supporting information from patient-specific risk factors and CTA examination could be supplied to improve the classification decision. We believe that this would help in reducing the relatively large number of false negatives detected from the test dataset. One such approach was introduced in^5^, who showed integration of quantitative perfusion and clinical data improves diagnostic performance. An integrated approach using ML-based analysis for predictors of ischemia such as in^30^ with the polar maps could be one approach. For SPECT, such approach integrating clinical, stress testing and MPI imaging data has shown to improve prediction performance^31^.

In the future, we also intend to investigate if the performance is improved by giving out the actual measured myocardial blood flow from each segment as an input for classification. For example, in the work of Betancur et al.^7^, the authors used quantitative polar maps from SPECT Tc-99 perfusion to improve classification performance. This would allow to evaluate the classification performance prospectively in Carimas software and also allow the potential clinical applications of the developed classifier to be thoroughly assessed. Another interesting approach would be to investigate if the classification could be performed directly on dynamic PET time-series. This would be very challenging due to the data containing time-wise changing physiologic and image characteristics and also due to the extent of the data, containing 24 time series of volumetric PET data. Currently, the authors are not aware if such approach exists.

Moreover, recent studies have shown the usefulness of automating the entire perfusion analysis pipeline in perfusion MRI imaging^32,33^. Naturally, a possible continuation study would be to implement such a pipeline for O-15 H2O MPI as well. This would require to implement automatic: (a) myocardial delineation and ROI drawing, (b) delineation of the blood pool and right ventricle, (c) kinetic modelling based on the extracted time-activity curves from blood input and tissue and finally (d) visualization and analysis of the polar map. Automatic data processing methods such as motion correction might also be necessary prerequisites. Although quite a challenging and extensive work to implement, we hypothesize that such approach would be eventually be very valuable nevertheless for O-15 H_2_O MPI and other PET perfusion tracers.

A limitation of our study is that only polar maps from pharmacological stress imaging were available for training and evaluation of the CNN. This is mainly due to low number of subjects imaged with rest perfusion imaging at our clinical imaging center. Thus, an evaluation of the classifier performance with rest datasets needs to be performed separately as an extension of this study. The data was also collected during 2007–2011 on using only one PET/CT system and only one perfusion tracer. Thus, we need to investigate the classifier performance also on datasets collected with different and more recent PET/CT systems and different tracers as an extension of this study, as well as study of the methodology extension to multi-modality datasets including SPECT, CT and MRI perfusion. We also applied a single analysis program to create the polar maps, which will limit its applicability to Carimas at the moment.

We also assumed that the location of the reduced perfusion corresponds to a location of ischemia rather than a previous myocardial infarction. This assumption was due to the collection of the patient cohort, where patients with previously known myocardial infarction were excluded from the prospective trial. However, the presence of infarction cannot be excluded entirely by stress myocardial blood flow alone, as this does not rule out cases where the patient has had an underlying myocardial infarction before undergoing perfusion imaging.

An additional limitation of our study is that we currently have only 138 patients at our disposal. This limits the use of our approach for per-vessel classification, as the group size is too small to create a meaningful and reliable division to per-vessel datasets for CNN training and testing. In comparison, the subject group sizes have been 957 subjects in^4^, 1160 and 1638 subjects in^7,8^, 946 polar maps in^9^ and 1980 subjects in^31^, when per-vessel classification has been applied. Although data augmentation could be implemented, we did not see this as applicable, as the polar maps are saved in a fixed coordinate space in Carimas software. Thus, implementing random rotations, translations or shifts in the polar maps would not be justifiable in our case. Augmentations to polar maps saved in non-fixed coordinate space would be feasible to implement, as the authors did in^11^, by using random rotations of maximum angle of 70 degrees. This could result in increase in performance.

## Conclusions

In conclusion, we’ve shown the feasibility of using a custom 2D CNN architecture to classify patients with suspected obstructive CAD as non-ischemic or ischemic based on polar map JPEG images alone in ^15^O–H_2_O MPI. The 2D CNN performance was close to the classification by clinical interpretation and a good agreement between the CNN and the clinical interpretation was reached in all but two cases.

However, further work is needed in investigating whether classification accuracy can be improved to the same level, if not superior to the clinical interpretation. Future work will concentrate on improving on the classification performance with addition of quantitative information, contextual patient data and evaluation of method generalizability across various datasets.

## Supplementary Information

Supplementary Information.

## Data Availability

The datasets generated during and/or analysed during the current study are available from the corresponding author on reasonable request.

## Code availability

The python source codes with the trained weights, or parameters, for the model generated during the current study are available from the corresponding author on a reasonable request and will be made available at the University of Turku GitLab portal of the corresponding author (https://gitlab.utu.fi/jarmo.teuho).
