# Supplementary material for: Classification of ischemia from myocardial polar maps in 15O–H2O cardiac perfusion imaging using a convolutional neural network
Source: Sci Rep. 2022 Feb 18;12:2839. doi: 10.1038/s41598-022-06604-x (PMC8857225; doi:10.1038/s41598-022-06604-x)
Supplement: Supplementary file 1 — Supplementary Information. [file 41598_2022_6604_MOESM1_ESM.pdf]

SUPPLEMENTARY INFORMATION FILE

Classification of ischemia from myocardial polar maps in $^{15}O\text{-}H_2O$ cardiac perfusion imaging using a convolutional neural network

Jarmo Teuho*[1,2,3], Jussi Schultz[3], Riku Klén[2,3], Juhani Knuuti[3], Antti Saraste[3,4], Naoaki Ono[1,5], Shigehiko Kanaya[5]

Affiliations:

1 Data Science Center, Nara University of Science and Technology, Nara, Japan

2 Turku PET Centre, University of Turku, Turku, Finland

3 Turku PET Centre, Turku University Hospital, Turku, Finland

4 Heart Centre, Turku University Hospital and University of Turku, Turku, Finland

5 Department of Science and Technology, Nara University of Science and Technology, Nara, Japan

SUPPLEMENTARY DATA 1 – EQUATIONS USED FOR CALCULATION OF CLASSIFICATION ACCURACY METRICS

Accuracy (ACC), F1 score (F1S), sensitivity (SEN), specificity (SPE) and precision (PRE) were defined as:

$$\mathrm{ACC} = \frac{\mathrm{TP+TN}}{\mathrm{TP+TN+FP+FN}}, \qquad (1)$$

$$\mathrm{F1S} = \frac{\mathrm{2TP}}{\mathrm{2TP+FP+FN}}, \qquad (2)$$

$$\mathrm{SEN} = \frac{\mathrm{TP}}{\mathrm{TP+FN}}, \qquad (3)$$

$$\mathrm{SPE} = \frac{\mathrm{TN}}{\mathrm{TN+FP}}, \qquad (4)$$

$$\mathrm{PRE} = \frac{\mathrm{TP}}{\mathrm{TP+FP}}, \qquad (5)$$

where TP, TN, FP and FN are the amount of true positives, true negatives, false positives and false negatives according to the predicted binary labels versus the reference labels from coronary artery angiography.

The net benefit was calculated from the test data as:

$$\text{Net benefit} = \frac{\mathrm{TP}}{\mathrm{N}} - \frac{\mathrm{FP}}{\mathrm{N}} \times \frac{\mathrm{p}_t}{1-\mathrm{p}_t}, \qquad (6)$$

where N is the total sample size in the test set (N=46) and p_t is the threshold probability to define when the patient is positive.

Cohen's Kappa coefficient κ was calculated as follows:

$$\kappa = \frac{p_o - p_e}{1-p_e}, \qquad (7)$$

where p_o is the relative observed agreement among raters and p_e is the hypothetical probability that the agreement happened by chance.

SUPPLEMENTARY DATA 2 – SUBJECT POLAR MAPS CLASSIFIED AS FALSE NEGATIVES SYSTEMATICALLY BY THE CNN

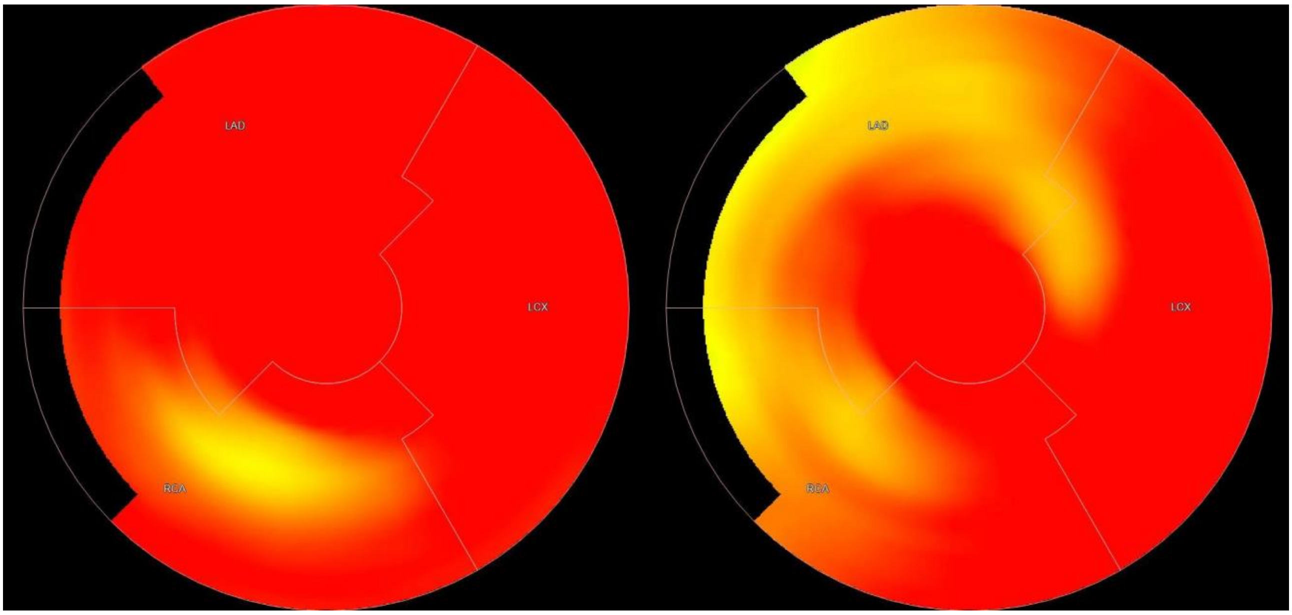

Supplementary Figure S1. Polar maps from two subjects classified systematically as FN by the CNN. The 1st subject was classified 90 times out of 100 as FN whereas the 2nd subject was classified 88 times out of 100 as FN. Visually, it is hard to determine whether the subjects have significant reduction of perfusion, although based on invasive coronary artery angiography both subjects were ischemic.

SUPPLEMENTARY DATA 3 – ACCURACY (ACC) AS OPTIMIZATION METRIC

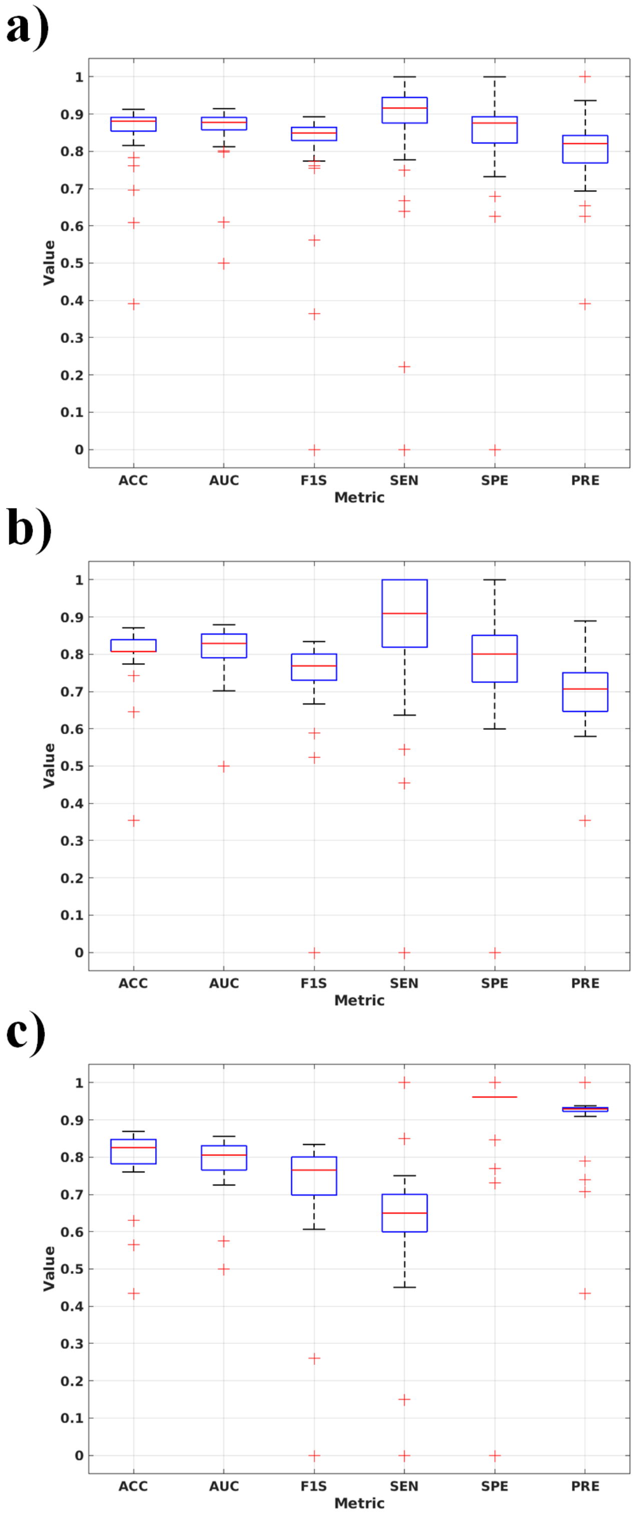

Supplementary Figure S2. CNN classification accuracy with a) training b) validation and c) test data. ACC = accuracy, AUC = area under the receiver operating characteristic curve, F1S = F1 score, SEN = sensitivity, SPE = specificity, PRE = precision.

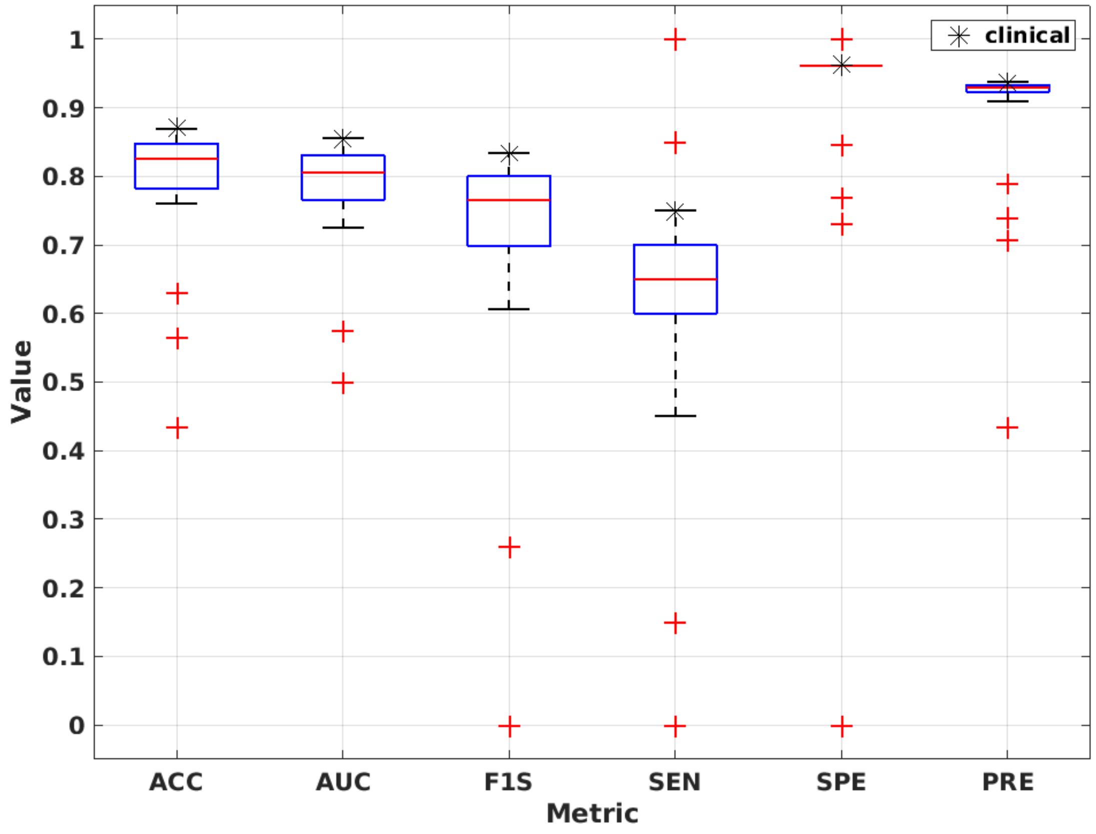

Supplementary Figure S3. CNN classification performance from 100 runs of the network versus the clinical interpretation. ACC = accuracy, AUC = area under the receiver operating characteristic curve, F1S = F1 score, SEN = sensitivity, SPE = specificity, PRE = precision.

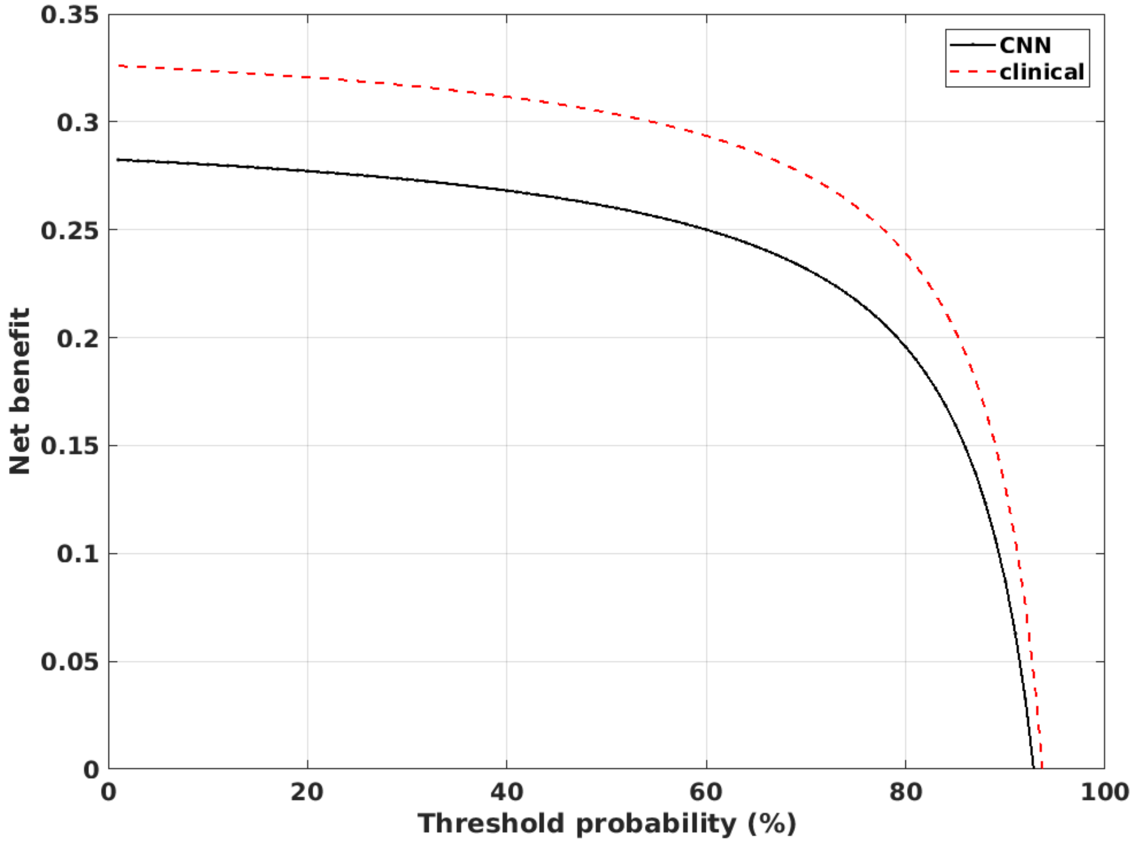

Supplementary Figure S4. Decision curve with net benefit for all threshold probabilities from 0 % to 100 % for CNN median over 100 runs of data and the clinical interpretation using the test dataset. Only positive values in the range of 0 to 1 for the net benefit are shown.

SUPPLEMENTARY DATA 4 – COMPARISON OF DIFFERENT CNN ARCHITECHTURES

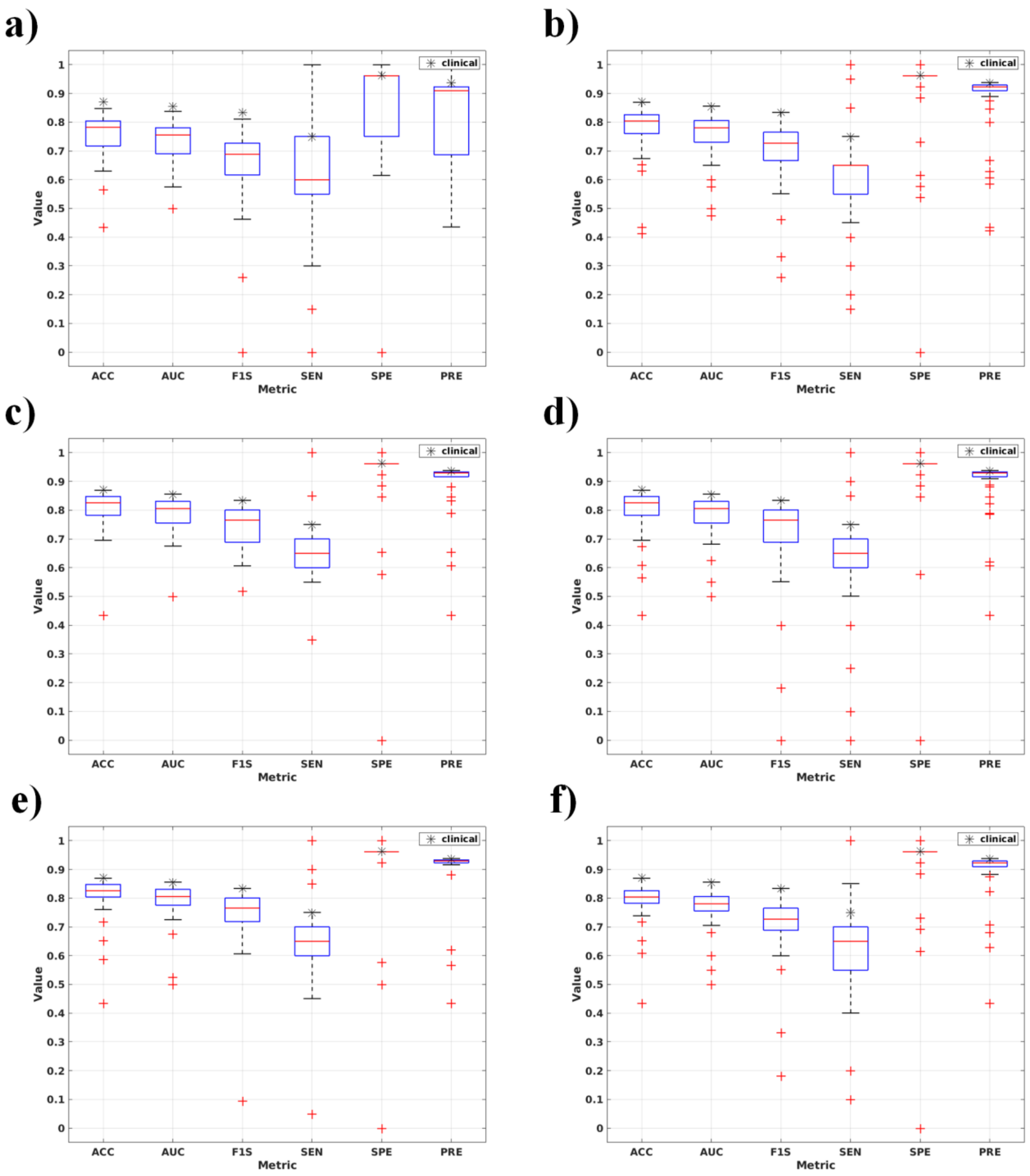

Supplementary Figure S5. CNN classification performance with changes introduced in the network architecture from 100 runs of the network versus the clinical interpretation. a) CNN without max-pooling operations, b) CNN with 3 convolutional layers, c) CNN with one deep layer, d) CNN with three deep layers, e) CNN with increased kernel size in first layer, f) CNN with increased kernel size in last layer. ACC = accuracy, AUC = area under the receiver operating characteristic curve, F1S = F1 score, SEN = sensitivity, SPE = specificity, PRE = precision.
